# Lipopolysaccharide‐induced inflammation does not alter muscle spindle afferent mechanosensation or sensory integration in the spinal cord of adult mice

**DOI:** 10.14814/phy2.13812

**Published:** 2018-09-03

**Authors:** Dasha Zaytseva, Anusha Allawala, Joy A. Franco, Shea Putnam, Adam M. Abtahie, Nina Bubalo, Connor R. Criddle, Tuan A. Nguyen, Peter Nguyen, Shreejit Padmanabhan, Puneet Sanghera, Martina Bremer, Tzvia Abramson, Katherine A. Wilkinson

**Affiliations:** ^1^ Department of Biological Sciences San José State University San Jose California; ^2^ Abbvie Biotherapeutics Redwood City California; ^3^ Department of Mathematics & Statistics San José State University San Jose California

**Keywords:** H reflex, inflammation, lipopolysaccharide, muscle spindle afferent

## Abstract

Inflammation is known to alter nervous system function, but its effect on muscle spindle afferent mechanosensation and sensory integration in the spinal cord has not been well studied. We tested the hypothesis that systemic inflammation induced by an intraperitoneal injection of the endotoxin lipopolysaccharide (LPS; 7.5 × 10^5^ endotoxin units/kg 18 h before experiment) would alter muscle spindle afferent mechanosensation and spinal cord excitability to Group Ia input in male and female adult C57Bl/6 mice. LPS injection caused a systemic immune response, evidenced by decreased white blood cell, monocyte, and lymphocyte concentrations in the blood, increased blood granulocyte concentration, and body weight loss. The immune response in both sexes was qualitatively similar. We used an in vitro muscle‐nerve preparation to assay muscle spindle afferent response to stretch and vibration. LPS injection did not significantly change the response to stretch or vibration, with the exception of small decreases in the ability to entrain to high‐frequency vibration in male mice. Similarly, LPS injection did not alter spinal cord excitability to Group Ia muscle spindle afferent input as measured by the Hoffman's reflex test in anesthetized mice (100 mg/kg ketamine, 10 mg/kg xylazine). Specifically, there were no changes in M or H wave latencies nor in the percentage of motor neurons excited by electrical afferent stimulation (H_max_/M_max_). Overall, we found no major alterations in muscle proprioceptor function or sensory integration following exposure to LPS at a dose and time course that causes changes in nociceptor function and central processing.

## Introduction

Systemic and local inflammation alter both peripheral and central nervous system function. Gram negative bacterial infections cause inflammatory pain through both direct and indirect action on nociceptors (Ji et al. 2016; Pinho‐Ribeiro et al. 2017). The endotoxin lipopolysaccharide (LPS) directly binds to toll‐like receptor 4 (TLR4) on nociceptors and triggers sensitization (Diogenes et al. 2011). LPS also activates immune and glial cells, causing the release of cytokines and reactive oxygen species, contributing to nociceptor sensitization and maintenance of inflammatory pain (Maier et al. 1993; Cunha et al. 2005; Binshtok et al. 2008; Silva 2010; Ji et al. 2016). Local addition of inflammatory factors can sensitize the Group III/IV muscle nociceptors to mechanical stimulation (Mense 1981; Hoheisel et al. 2005). Similarly, inflammation is known to alter central nervous system excitability, contributing to hyperalgesia (Dubner and Ruda 1992; Watkins et al. 1995), an increased risk of seizures (Riazi et al. 2008), and decreased performance on memory tasks (Arai et al. 2001; Chen et al. 2008).

The effect of inflammation on mechanosensation of the Group Ia/II muscle spindle afferents and central integration of proprioceptive sensory information is not well understood. Gait abnormalities and motor deficits are seen 10–24 h following LPS exposure in rats (Brown et al. 1997), and alterations in muscle spindle afferent signaling or integration could contribute to these deficits. There are multiple pathways by which inflammatory factors could alter muscle proprioceptor function. Dorsal root ganglion cells of all sizes, including large diameter afferents, express TLR4 (Barajon et al. 2009). The activity of both the ion channels shown to be involved in muscle spindle afferent mechanotransduction, Piezo2 (Woo et al. 2015) and ASIC3 (Lin et al. 2016), can be modulated by inflammatory factors (Jones et al. 2005; Li et al. 2010; Dubin et al. 2012). Reactive oxygen species, which are produced following bacterial infection or LPS exposure (Silva 2010), increase the sensitivity of muscle spindle afferents to muscle movement (Delliaux et al. 2009). Bradykinin also increases muscle spindle afferent sensitivity to stretch (Wenngren et al. 1998), an effect at least partially caused by changes in gamma motor neuron drive (Pedersen et al., 1997).

In this study, we tested the hypothesis that a systemic immune challenge could alter the sensitivity of muscle spindle afferents to muscle movement as well as increase spinal cord excitability to Group Ia muscle spindle afferent sensory input. We chose an intraperitoneal injection of a high but sub‐septic dose of LPS to induce a systemic immune response (Galanos et al. 1979; Kozak et al. 1994). An in vitro mouse muscle‐nerve preparation was used to assay muscle spindle afferent responsiveness to muscle stretch and vibration (Wilkinson et al. 2012). Motor neuron excitability in response to Group Ia muscle spindle afferent input was assessed using the Hoffman's or H Reflex technique, which is the electrophysiological analog of the muscle stretch reflex. Adult mice of both sexes were tested as there are well‐described sex differences in the immune system (Klein 2000). Unlike the robust changes in nociceptor sensory responses and central processing of nociceptive sensory input found 30 min to 24 h following similar doses of LPS (Maier et al. 1993; Brown et al. 1997), we found no major changes following LPS‐induced inflammation in muscle proprioceptor function or motor neuron excitability to muscle spindle afferent sensory information.

## Methods

### Animals

All procedures were approved by the Institutional Animal Care and Use Committee at San José State University (Protocols #990 and 1011). Seven‐ to ten‐week‐old adult male (*n* = 75) and female (*n* = 72) C57Bl/6 mice were purchased from Simonsen Laboratories (Gilroy, CA, USA). Mice were housed in cages of 4–8 mice under a 12:12 h light‐dark cycle and fed standard laboratory chow and water ad libitum. Visual health and activity checks were performed daily by trained animal care staff and cages changed twice a week. Eighteen hours before sacrifice, mice were given a 200 *μ*L intraperitoneal (IP) injections of saline (SAL) or lipopolysaccharide (LPS from *Escherichia coli* O111:Br; 7.5 × 10^5^ endotoxin units/kg LPS in saline, equivalent to 0.5 mg/kg LPS for lots 012M4098V & 122M4001V used in this study; Millipore Sigma, Darmstadt, Germany). Mice were weighed prior to injection and again at the start of the experiment. A dish of moistened chow was placed on the floor of the cage following LPS injections to encourage eating. Mice were used in one of three experiments as described below.

### Blood collection for immune cell quantification by flow cytometry

**Figure 1:**
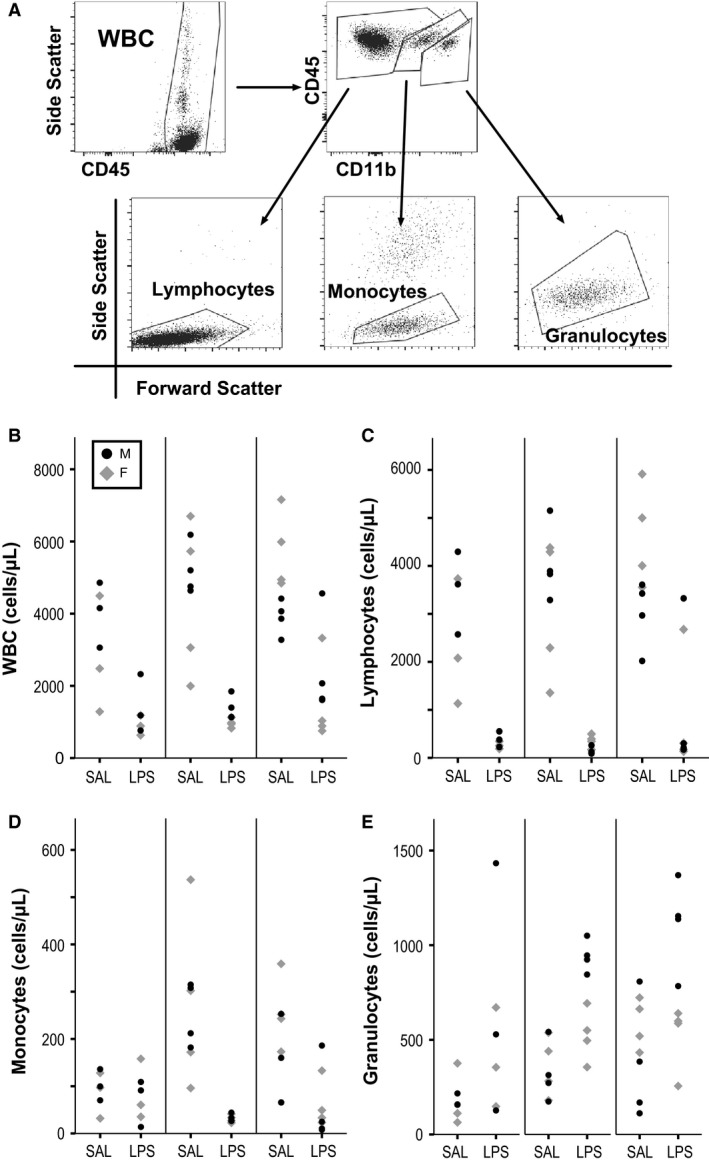
Immune cell quantification with flow cytometry. (A) Gating strategy used to identify total white blood cells (WBC), lymphocytes, monocytes, and granulocytes in blood samples. Blood concentrations (cells/*μ*L) of WBC (B), lymphocytes (C), monocytes (D), and granulocytes (E) from SAL and LPS animals in three different experiments. Each point is an individual animal with males denoted as black circles and females as gray diamonds.

Approximately 18 h after injection, mice were deeply anesthetized with isofluorane (5% in oxygen), blood was collected by cardiac puncture, and the animals immediately decapitated. We also dissected out the spleen from most mice and determined the wet weight. We performed three rounds of blood collection, with 3 (Experiment 1) or 4 (Experiments 2 & 3) mice per group (male SAL, male LPS, female SAL, female LPS) each time for a total of 44 mice. In Experiments 2 and 3, mice were also given injections of saline twice a week for 4 weeks prior as they were collected with additional groups testing chronic exposure to LPS (results not shown). The blood was stored in heparin tubes, vortexed, and stored at 4°C up to 48 h until staining. 50 *μ*L of the anticoagulated blood was transferred into a Trucount tube (BD Biosciences, San Jose, CA, USA). For each sample, 20 *μ*L of antibody cocktail was added to the Trucount tube and vortexed (CD11b FITC clone M1/70 and CD45 PerCP/Cy5.5 clone 30‐F11; Biolegend, La Jolla, CA, USA). The antibody cocktail was prepared in a phosphate buffered saline staining buffer containing 10% fetal bovine serum and 0.09% sodium azide. The samples were incubated in the dark for 30 min at room temperature. After incubation, 450 *μ*L of 1X FACS Lysing Solution (BD Biosciences) was added to each tube and vortexed to lyse the red blood cells. The samples were then incubated in the dark for 15 min at room temperature. The gating strategy is described below in Figure 1A and adapted from the literature (Weaver et al. 2010). For each sample, 30,000 events were recorded using either the FACS Calibur or FACS Canto Flow Cytometer (BD Biosciences). Data analysis was performed using Flowjo software (Flowjo, Ashland, OR, USA). The number of beads in the Trucount tube were also recorded and used to calculate the concentration of the immune cells according to the manufacturer's instruction. White blood cells, lymphocytes, granulocytes, and monocytes were quantified as cells/*μ*L.

### Electrophysiological recording and analysis of muscle spindle afferent activity

Extracellular recording of muscle sensory neuron activity was performed using an in vitro isolated muscle‐nerve preparation described in detail previously (Wilkinson et al. 2012; Franco et al. 2014). Mice were placed in an induction chamber, deeply anesthetized with isoflurane (5% in oxygen), decapitated, and skinned. The spleen of most animals was removed and weighed. The legs were placed in an oxygenated (95% O_2_/5% Co_2_) tissue bath filled with chilled low calcium, high magnesium artificial cerebrospinal fluid (aCSF), containing in mM: 128 NaCl, 1.9 KCl, 1.2 KH_2_PO_4_, 26 NaHCO_3_, 0.85 CaCl_2_, 6.5 MgSO_4_, and 10 glucose (pH 7.4 ± 0.5), and perfused with 95%O_2_/5% CO_2_. The extensor digitorum longus (EDL) muscle and deep peroneal branch of the sciatic nerve were dissected and placed into an oxygenated (100% O_2_) tissue bath filled with 24°C synthetic interstitial fluid (SIF) containing in mM: 123 NaCl, 3.5 KCl, 0.7 MgSO_4_, 1.7 NaH_2_PO_4_, 2.0 CaCl_2_, 9.5 NaC_6_H_11_O (sodium gluconate), 5.5 glucose, 7.5 sucrose, and 10 *N*‐2‐hydroxyethylpiperazine‐*N′*‐2‐ethanesulfonic acid (HEPES) (pH 7.4 ± 0.5). The tendons of the EDL muscle were secured with 5‐0 nylon thread to a stationary tissue post on one end and the lever arm of a dual force and length controller and transducer on the other end (300C‐LR, Aurora Scientific, Inc.; Aurora, ON, Canada).

The length at which maximal twitch contractile force was produced, or optimal length (*L*_o_), was determined using bath electrodes (0.5 msec pulse width, supramaximal voltage; S88 Stimulator, Grass Technologies; San Carlos, CA, USA). Muscles were held at this length throughout the experiment. The cut end of the sciatic nerve was suctioned into a bipolar glass micropipette (tip diameter of 60–100 *μ*m) housing an electrode connected to an extracellular amplifier with headstage (Model 1800, A‐M Systems; Sequim, WA, USA). Neural responses to a battery of ramp and hold stretches and sinusoidal vibrations were then digitized and recorded (PowerLab, ADInstruments; Sydney, Australia). The muscle was stretched to three physiological lengths (2.5%, 5%, and 7.5% of *L*_o_; 40% *L*_o_/sec ramp speed). Each stretch was repeated three times with a 1 min rest period in between to prevent muscle thixotropic effects. Sinusoidal vibrations 9 sec in length were performed at four frequencies (10, 25, 50, and 100 Hz) and four amplitudes (5, 25, 50, and 100 *μ*m) with 1 min rest in between, for a total of 16 different vibrations. In a subset of muscles, the maximal tetanic contractile force was measured (500 msec train, 120 Hz, 0.5 msec pulse width). Wet weight of the EDL was determined and cross sectional area (CSA) calculated as (mass)/(*L*_o_ × density), using 1.06 kg/L for muscle density (Brooks and Faulkner 1988).

The Spike Histogram function of Lab Chart (ADInstruments) was used to identify individual afferents based on spike shape. A total of 79 muscle spindle afferents were used in this study (23 male SAL from 16 mice, 18 male LPS from 14 mice, 17 female SAL from 11 mice, 21 female LPS from 13 mice). Due to technical reasons, responses to both stretch and vibration were not recorded for all afferents and the numbers used for each test are denoted in the relevant figures. Instantaneous firing frequency was measured at baseline (BL), at the beginning of the hold portion of the stretch (0.5 sec after ramp completed, initial static time or IST), and during the plateau phase of stretch (0.5 sec before end of stretch, final static time or FST). The peak firing during the ramp phase was also measured (dynamic peak or DP). Values were averaged for the three repeats of each stretch as no systematic order effect was observed. Dynamic Index (DI) was calculated as DP‐IST. For each vibration we determined if the afferent could entrain by firing at the same time during each cycle of vibration. Because all included afferents displayed the characteristic adaptation of firing frequency during the hold phase of stretch typical of muscle spindle afferents and Group Ib Golgi Tendon Organ afferents rarely discharge to stretches of the lengths given (Houk and Henneman 1967), we likely only included muscle spindle afferents in our sample.

Maximal tetanic contraction force normalized to muscle CSA was calculated for a subset of muscles and compared with previously reported values to confirm muscle health (Brooks and Faulkner 1988). Muscle tension at baseline, peak tension during stretch, and plateau tension immediately before stretch was released were determined at the 5% *L*_o_ stretch length. We calculated the parallel (*E*_PE_) and series modulus of elasticity (*E*_SE_) normalized to muscle CSA using the following formula from (Wolff et al. 2006):

$$\begin{array}{cc} E & =\frac{\Delta F}{\underline{\mathrm{CSA}}}\\ & \;\;\frac{\Delta L}{L_{o}} \end{array}$$

*Equation 1*: Modulus of elasticity. ∆*F* is the difference in tension (mN) from baseline; ∆*L* is the change in length (mm) from *L*_o_. ∆*F* measured from baseline to peak of stretch for *E*_SE_ and end of stretch for *E*_PE_.

### Hoffman's (H) reflex testing to determine motor neuron excitability

Motor neuron excitability to Group Ia muscle spindle afferent sensory input was measured using the electrophysiological Hoffman's or H‐reflex test on a total of 52 mice (13 male SAL, 12 male LPS, 14 female SAL, 13 female LPS). Mice were anesthetized by an intraperitoneal injection of ketamine (100 mg/kg) and xylazine (10 mg/kg). Anesthesia type and level can alter spinal reflexes, however, H reflex excitability under ketamine/xylazine was found to be similar to awake reflexes (Ho and Waite 2002). A rectal temperature probe (BAT‐12, Physitemp; Clifton, NJ, USA) was used to monitor animal body temperature throughout the experiment. The flow of warm water through the surgical base plate was changed as necessary to maintain adequate body temperature. Depth of anesthesia was monitored by testing for a response to toe pinch at least once every 10 min and anesthesia re‐administered as necessary. A thigh incision was made to expose the sciatic nerve and allow placement of a tungsten bipolar stimulating electrode connected to a stimulator (S44 Stimulator, Grass Technologies). Bipolar tungsten recording electrodes were pushed through the skin to contact the 4th dorsal interossei muscle of the foot and connected to an extracellular amplifier with headstage (Model 1800, A‐M Systems). Following stimulation of the sciatic nerve a short latency M wave was recorded as a result of direct motor neuron stimulation and a longer latency H wave from reflex activation of motor neurons via Group Ia afferents. Preliminary trials determined that more proximal leg muscles, including the EDL, had M and H wave latencies that were too close together to reliably distinguish the two waves. H wave threshold was determined and a 0.1 Hz train of 20 stimuli (0.2 ms pulse width) was given at up to nine stimulus strengths (T, 1.3T, 1.5T, 2T, 3T, 5T, 6T, 7T, and 8T).

Amplified neural data were digitzed using an analog to digital board (PowerLab, ADInstruments) and analyzed using LabChart software (ADInstruments). H and M amplitudes and latencies were measured at all stimulus strengths following 0.1 Hz stimulations. To allow for comparison between subjects, the maximum H wave amplitude was divided by the maximum M wave amplitude (H_max_/M_max_) to express the percentage of motor neurons activated by Group Ia sensory input and compared between treatment groups.

### Statistics

Since female body weights were significantly lower than male body weights, we used an Independent samples *t*‐test to compare body weight changes in LPS versus SAL control groups for each sex. To account for day to day differences in staining and other technical issues, we fit a three factor ANOVA to compare WBC, lymphocyte, monocyte, and granulocyte blood concentrations (date of experiment, drug (LPS or SAL), sex). BL, IST, FST, DP, and DI were compared using a three factor ANOVA model, with stretch length, sex, and drug as factors. *E*_PE_, *E*_SE_, body weight change, spleen weight, muscle weight, *L*_o_, CSA, maximal tetanic contraction strength, H and M Latencies, and H_max_/M_max_ were compared with a two factor ANOVA model (sex and drug). Ability to entrain to vibration was compared between groups using a multiple logistic regression model with vibration amplitude, vibration frequency, sex, and drug as factors. Since we did observe a sex difference we fitted individual logistic regression models for each sex with amplitude, frequency, and drug as factors. Values are given as mean ± SEM, error bars on graphs indicate standard error of the mean, and all differences are considered significant if *P* < 0.05.

## Results

### Confirmation of LPS‐induced inflammation

Eighteen hours post LPS injection, both male and female mice lost a significant amount of weight compared to SAL controls (SAL: 0.03 ± 0.07 g, *n* = 73; LPS: −1.98 ± 0.09 g, *n* = 74; 2 factor ANOVA, main effect of condition *P* < 0.001; main effect of sex *P* = 0.79; sex × condition interaction *P* = 0.93; Table 1).

**Table 1:** Body and spleen weight changes following LPS

Condition	Body weight change (g)	Spleen weight (mg)
M SAL	0.01 ± 0.10 (38)	56 ± 1 (18)
M LPS	−1.99 ± 0.15* (37)	71 ± 2* (19)
F SAL	0.05 ± 0.10 (35)	68 ± 2 (13)
F LPS	−1.97 ± 0.09* (37)	74 ± 3 (15)

Group averages ± standard error of the mean. Group *n* shown in (). *Denotes *P* < 0.05 from SAL control of same sex.

Spleen weight significantly increased following LPS treatment (2 factor ANOVA; main effect drug *P* < 0.001), however there was a significant sex by drug interaction, so we compared spleen weights of the two sexes individually (main effect sex *P* = 0.001; sex × drug interaction *P* = 0.046). Spleen weight significantly increased in males but not females following LPS treatment (Independent samples *t*‐test; male *P* < 0.001; female *P* = 0.11; Table 1).

LPS treatment led to robust decreases in WBC and lymphocyte blood concentrations in both male and female mice (3 factor ANOVA; main effect drug *P* < 0.001 for both; main effect sex *P* = 0.29 and 0.73; sex*drug interaction *P* = 0.43 and 0.81; Fig. 1B–C). Monocyte concentrations were also significantly decreased following LPS in both sexes, although there was more variability between the three experiments (main effect drug *P* < 0.001 for both; main effect sex *P* = 0.36; sex*drug interaction *P* = 0.46; Fig. 1D). Granulocyte levels were significantly increased following LPS treatment (*P* < 0.001), however the changes were larger in male mice (main effect sex *P* = 0.025; sex*drug interaction *P* = 0.001; Fig. 1E). Overall, the LPS challenge led to significant changes in immune cell concentrations in the blood suggesting our treatment was sufficient to cause a robust systemic immune response.

### Muscle properties including elasticity unchanged following LPS

Muscle anatomical properties and elasticity were measured for all muscles from which muscle spindle afferent response to stretch or vibration was assessed with the exception of two muscles from which an accurate muscle weight was not obtained (male SAL *n* = 16; male LPS *n* = 3; female SAL *n* = 6; female LPS *n* = 8). As expected, muscle mass, *L*_o_, and CSA were significantly lower in F mice (2 factor ANOVA; muscle mass: *P* < 0.001; *L*_o_: *P* = 0.003; CSA: *P* = 0.007). Treatment with LPS did not alter muscle mass, *L*_o_, or CSA in either sex (main effect of drug *P* = 0.40, 0.56, and 0.55; sex*drug interaction *P* = 0.15, 0.61, and 0.23). We found no effect of drug on muscle parallel or series modulus of elasticity (2 factor ANOVA; *E*_PE_: *P* = 0.41; and *E*_SE_: *P* = 0.27). There were no significant sex differences in these CSA normalized elasticity values, although female values trended lower (*E*_PE_: *P* = 0.92, sex*drug interaction *P* = 0.74; *E*_SE_: *P* = 0.16, sex*drug interaction *P* = 0.76; Table 2). Similarly, all groups exhibited maximal tetanic contraction strengths in the healthy range, with no effect of drug or sex difference (2 factor ANOVA; 21.7 ± 9.7 mN/cm^2^, *n* = 44; main effect of drug *P* = 0.35; main effect of sex *P* = 0.45; sex*drug interaction *P* = 0.61). Overall, muscle anatomical, elastic, and contractile properties were not significantly changed following LPS treatment.

**Table 2:** Muscle anatomical properties unchanged following LPS

Condition	Muscle mass (mg)	*L*_o_ (mm)	CSA (mm^2^)	*E*_PE_ (mN/mm^2^)	*E*_SE_ (mN/mm^2^)
M SAL (16)	9.3 ± 0.4	11.8 ± 0.2	0.74 ± 0.03	1.2 ± 0.2	1.7 ± 0.2
M LPS (13)	10.2 ± 0.5	12.1 ± 0.2	0.81 ± 0.05	1.3 ± 0.2	1.8 ± 0.3
F SAL (16)	8.0 ± 0.4	11.1 ± 0.3	0.69 ± 0.03	0.9 ± 0.1	1.3 ± 0.1
F LPS (18)	7.8 ± 0.3	11.1 ± 0.3	0.67 ± 0.03	1.1 ± 0.1	1.6 ± 0.2

Group averages ± SEM Group *n* shown in (). No significant effects of LPS on any measure. *F* values for muscle mass, *L*_o_, and CSA significantly lower than *M* values.

### Muscle spindle afferent response to stretch and vibration mostly unchanged following LPS

**Figure 2:**
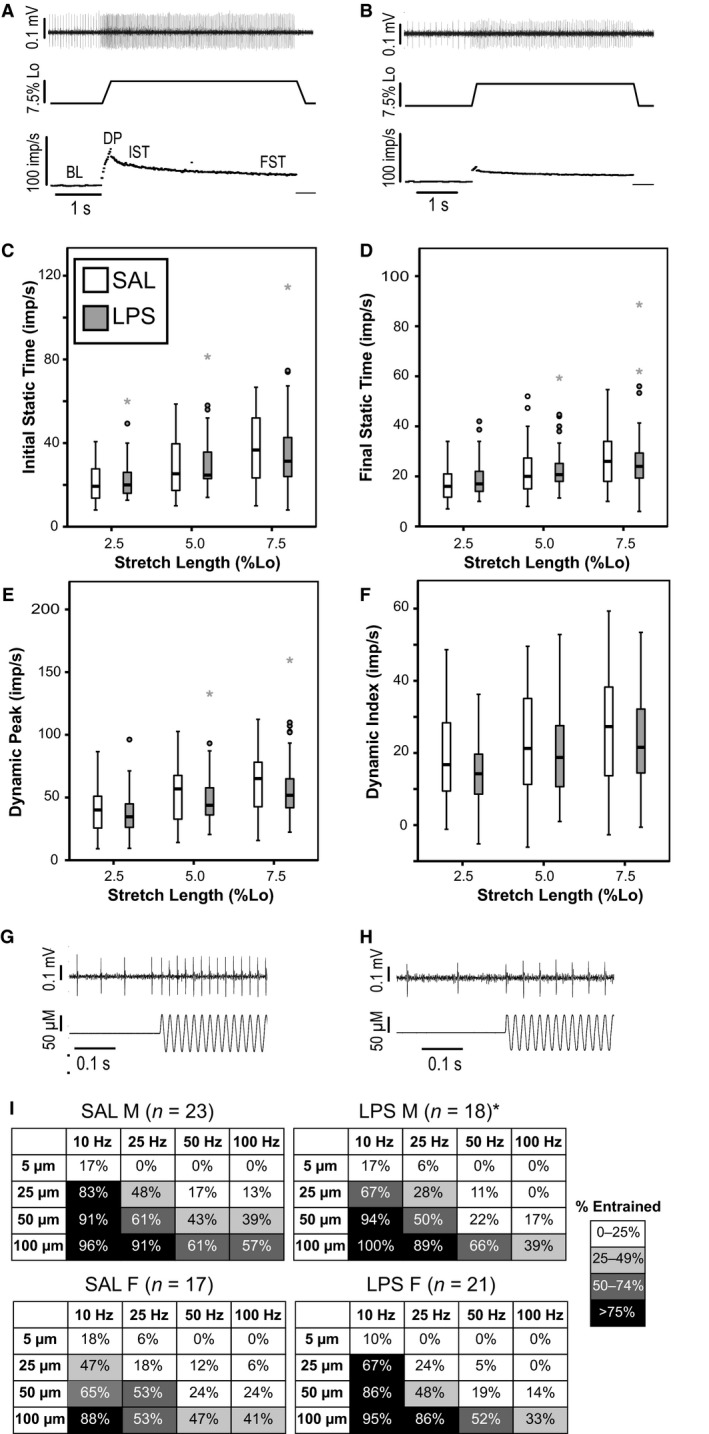
Effect of LPS on muscle spindle afferent response to stretch and vibration. Example ramp and hold stretch response in a M SAL (A) and M LPS (B) animal. Top trace is raw neural response, middle trace is the muscle length, bottom trace is the calculated instantaneous frequency. Points where baseline (BL), dynamic peak (DP), initial static time (IST), and final static time (FST) measured are denoted on instantaneous frequency trace. Group IST (C), FST (D), DP (E), and dynamic index (DI=DP‐IST; F) shown at all 3 stretch lengths. Data from both sexes were collapsed since there was no sex effect. There was a main effect of stretch length for all measures but no effect of LPS. Line indicates median, whiskers indicate 95% confidence interval, outliers denoted with circles, extreme outliers with a star. Example responses to a 50 *μ*m, 50 Hz vibration from the same M SAL (G) and M LPS (H) mice as shown in A & B. The M SAL animal entrained to vibration while the M LPS animal did not. Top trace is the raw neural response; bottom trace is muscle length. Percentage of animals in each group that could entrain to a given vibration (I). M and F animals are shown separately as there was a main effect of sex. Darker box color corresponds with a higher percentage of entrainment at a given vibration. Animals in all groups were more likely to entrain to a lower frequency (left columns) and higher amplitude (bottom rows) vibrations. M LPS groups were less likely to entrain to vibration, especially higher frequency vibrations, than M SAL (*P* = 0.02).

As expected, firing rate during the hold (IST, FST) and ramp (DP) phases of stretch significantly increased with increasing stretch length (3 factor ANOVA; main effect of stretch length *P* < 0.001 for all; Fig. 2A–E). LPS did not alter firing rates during any point of the stretch (IST *P* = 0.53; FST *P* = 0.50; D *P* = 0.56), with no sex differences (main effect of sex *P* = 0.64, 0.79, and 0.67; sex*drug interaction *P* = 0.30, 0.47, and 0.41). This suggests no changes in muscle spindle afferent static stretch sensitivity. Dynamic sensitivity was measured by DI during ramp and hold stretch and whether afferents could entrain to high frequency or low amplitude vibration. DI was not significantly different following LPS, although it was trending lower (main effect of drug *P* = 0.08; main effect of sex *P* = 0.82; sex*drug *P* = 0.77; Fig. 2F). As expected, muscle spindle afferents were less likely to entrain to higher frequency or lower amplitude vibrations (Binary linear regression; *P* < 0.001 for both). There was no effect of drug (*P* = 0.26), however there was a significant sex difference (*P* < 0.001). Since there was a sex difference we analyzed male and female mice separately. Both sexes were less likely to entrain to high frequency or low amplitude vibrations (Binary linear regression; *P* < 0.001 for all), but only male mice showed a decreased ability to entrain to vibration following LPS (male *P* = 0.02; female *P* = 0.44; Fig. 2G–I). Dynamic sensitivity was slightly decreased in male mice following LPS, but the biological relevance of this change in response to muscle movement is unlikely to be large.

### Spinal cord excitability as measured by the H Reflex unchanged following LPS

**Figure 3:**
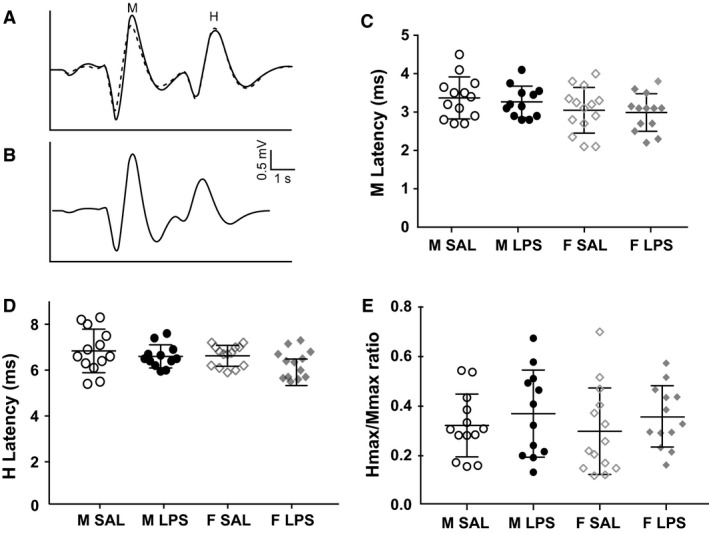
Effect of LPS on H Reflex Measurements. Example trace from a male saline injected (M SAL; A) animal and a M LPS (B) animal. Recordings at stimuli where maximum H (H_max_; solid line) and M (M_max_; dashed line) waves were recorded are overlaid for A. Both H_max_ and M_max_ were recorded at same stimulus voltage in B. M latency (C), H latency (D), and percentage of motor neurons activated by electrical Group Ia stimulation (H_max_/M_max_; E) were unaltered by LPS treatment in mice of either sex. Each point denotes an individual animal's value (M SAL *n* = 13; M LPS *n* = 12; F SAL *n* = 14; F LPS *n* = 13), line indicates mean, and error bars are ± SE.

Reflex responsiveness to Group Ia muscle spindle afferent sensory input was assayed using the H reflex. There was no effect of LPS on either the H or M wave latencies (main effect of drug *P* = 0.58 and 0.11, respectively; Fig. 3A–D). Similarly, the percentage of motor neurons that could be activated by electrical stimulation was unchanged with LPS (2 factor ANOVA; H_max_/M_max_; main effect of drug *P* = 0.22; Fig. 3E). Female M latency was significantly shorter than male M latency, however there was no sex by drug interaction (main effect of sex *P* = 0.043; sex × drug *P* = 0.88). No sex differences were observed in H latencies or H_max_/M_max_ (main effect of sex *P* = 0.14 and 0.67; sex × drug interaction *P* = 0.73 and 0.91). Overall, we found no LPS induced changes in H reflex excitability.

## Discussion

In this study, we found only minor changes in muscle spindle afferent mechanosensation in male mice and no changes in the spinal cord integration of Group Ia afferent sensory information following 18 h of LPS‐induced systemic inflammation. We confirmed that our dose of LPS induced a robust immune response, as evidenced by weight loss and changes in immune cell concentrations in the blood. At this dose of LPS after 8–24 h others have reported a similar decrease in weight, decreased food intake, and decreased locomotion, characteristic of LPS‐induced sickness behavior (Corona et al. 2010). The increased blood levels of granulocytes and decreased levels of white blood cells, lymphocytes, and monocytes are similar to what has been reported previously in male mice (Zager et al. 2012). Both male and female mice responded similarly to the immune challenge, but there was some evidence that the male immune response was larger. Only male mice exhibited significantly larger spleen weights following LPS, although female spleen weights were trending higher. Males also exhibited a larger increase in blood granulocyte concentration than female mice. Overall, we saw clear evidence of LPS‐induced inflammation in mice of both sexes 18 h after LPS administration.

We hypothesized that the muscle spindle afferent response to muscle movement following LPS would be increased, similar to what is seen in Group III/IV afferents sensitized to mechanical forces following inflammation (Mense 1981; Hoheisel et al. 2005), and that this effect could be mediated by changes to neural function or the mechanical forces seen by the spindle afferents. Potential mechanisms for inflammation induced changes to muscle spindle afferent mechanosensation could have included LPS acting on the TLR4 receptor (Barajon et al. 2009) or inflammatory factors altering the function of the mechanically sensitive ion channels Piezo2 or ASIC3 (Jones et al. 2005; Li et al. 2010; Dubin et al. 2012). Alternatively, inflammation could have altered the mechanical forces felt by the spindle afferents by altering intrafusal fiber stiffness through changes in extracellular matrix proteins (Sorokin 2010). We found no changes in muscle elasticity following LPS, however we did not directly measure intrafusal fiber elasticity which could vary independently from extrafusal fiber elasticity. Direct measures of intrafusal fiber elasticity in isolated muscle spindles would be necessary to confirm that LPS did not alter intrafusal fiber elasticity. Similarly, we observed no changes in the static response to stretch following LPS treatment, which supports the conclusion that the mechanical forces felt by the muscle spindle afferents had not changed following LPS. The only potential changes in mechanosensation following LPS were observed during dynamic stretch. Dynamic index was trending lower after LPS (*P* = 0.08) and afferents from male but not female mice showed a decreased ability to entrain to high‐frequency vibration (Fig. 2I). The functional significance of these changes is unlikely to be large, as overall firing rate of the muscle spindle afferents was largely unaltered. These changes in dynamic sensitivity in male mice could be suggestive of small changes in intrafusal fiber stiffness or changes in ion channel function, for instance faster inactivation of the Piezo2 channel (Dubin et al. 2012; Lewis et al. 2017). Direct recordings of ionic currents in muscle spindle afferents would be necessary to determine whether this level of systemic inflammation can alter channel function. As dynamic sensitivity only significantly changed in male mice and there was evidence of an increased immune response in male mice, it is possible that a larger or more prolonged inflammatory challenge is needed to cause more dramatic changes in muscle spindle afferent mechanosensation.

In the absence of changes to muscle spindle afferent mechanosensation, motor control could still be compromised by inflammation if spinal cord integration of sensory information was altered. We tested the hypothesis that the motor neurons would become more excitable in response to electrical Group Ia sensory stimulation using the H reflex test. Changes in motor neuron excitability in the absence of altered Group Ia response to stretch would suggest heightened reflex sensitivity, similar to what is seen in nociceptive reflexes following tissue injury or inflammation (Cook et al. 1986; Ferrell et al. 1988; Herrero et al. 1997). We found no evidence for increased spinal cord excitability to Group Ia sensory information as there were no effects of LPS on H latency or the proportion of motor neurons activated by afferent stimulation (H_max_/M_max_). The only sex effect in reflex measurements was a significantly shorter M latency in females. As females weighed significantly less than males, it is possible that their limb length and nerve lengths were also shorter and could explain the shorter M latencies. Our findings suggest that motor reflexes and behavior are unlikely to be compromised during a relatively short exposure to systemic inflammation.

Overall, we found only minor changes in muscle spindle afferent mechanosensation in male mice and no changes in reflex excitability to Group Ia sensory input in mice of either sex following LPS‐induced systemic inflammation. One limitation of our study, however, is that we only looked at a single time point following the LPS challenge (18 h). At this point, there was clear evidence of a robust systemic immune response, but it is possible we missed earlier changes in function that had recovered by the time of our measurement. Similarly, prolonged inflammation could lead to more severe effects. In any case, our results contrast with the clear changes in nociceptor function and central integration following similar inflammatory challenges and suggest that muscle spindle afferent sensory function is unaltered following relatively short exposure to inflammation.

## Data Files

All raw data used in the figures and tables can be found in the Supplementary Data file. Data are organized into labeled sheets by type.

## Conflict of Interest

The authors have no conflicts of interest to disclose.

## Data Accessibility
